# Attention Modulates Neural Responses to Unpredictable Emotional Faces in Dorsolateral Prefrontal Cortex

**DOI:** 10.3389/fnhum.2016.00332

**Published:** 2016-06-28

**Authors:** Guangming Ran, Xu Chen, Qi Zhang, Yuanxiao Ma, Xing Zhang

**Affiliations:** ^1^Faculty of Psychology, Southwest UniversityChongqing, China; ^2^Research Center of Mental Health Education, Southwest UniversityChongqing, China; ^3^School of Education Science, Guizhou Normal UniversityGuiyang, China

**Keywords:** emotional faces, unpredictability, attention, social cues, dorsolateral prefrontal cortex

## Abstract

Unpredictability about upcoming emotional events disrupts our ability to prepare for them and ultimately results in anxiety. Here, we investigated how attention modulates the neural responses to unpredictable emotional events. Brain activity was recorded using functional magnetic resonance imaging (fMRI) while participants performed a variation of the emotional task. Behaviorally, we reported a fear-unpredictable effect and a happy-unpredictable effect. The fMRI results showed increased activity in the right dorsolateral prefrontal cortex (dlPFC) for unpredictable fear faces (Experiment 1) and decreased activity in the left dlPFC for unpredictable happy faces (Experiment 2) when these faces were unattended, probably reflecting that unpredictability amplifies the negative impact of fear faces and reduces the positive impact of happy faces. More importantly, it was found that the right dlPFC activity to unpredictable fear faces was diminished (Experiment 1) and the left dlPFC activity to unpredictable happy faces was enhanced (Experiment 2) when these faces were attended. These results suggest that attention may contribute to reducing the unpredictability about future emotional events.

## Introduction

Knowledge about upcoming emotional events can be helpful in preparing for such events (Barbalat et al., 2012; Ran et al., 2014). However, there is often unpredictability about whether an upcoming emotional event will actually occur, which interferes with our daily routines, and ultimately affects the maintenance of mental health (Singer et al., 2009; Jin et al., 2013). An increasing number of studies have shown that unpredictability or uncertainty increases individuals' anxiety and fear (Bredemeier and Berenbaum, 2008; Grupe and Nitschke, 2013).

Considerable research has investigated the neural substrates of unpredictability, using paradigms examining decision-making (Volz et al., 2003, 2004, 2005; Bach et al., 2009), perceptual matching (Walsh and Phillips, 2009), pain expectation (Brown et al., 2008), and emotional expectation (Nitschke et al., 2006; Herwig et al., 2007). For example, evidence from functional MRI data suggests that prefrontal cortices are among the brain regions implicated in processing emotional unpredictability (Nitschke et al., 2006; Herwig et al., 2007; Schienle et al., 2010). More importantly, there is evidence that the dorsolateral portion of the prefrontal cortices (dlPFC) plays a critical role in the cognitive control of emotional unpredictability (Herwig et al., 2007; Aupperle et al., 2012).

Recent Bayesian models of perception have proposed that attention improves the precision of our inferences (Rao, 2005; Friston, 2009), suggesting that it may contribute to reducing the unpredictability of upcoming events. This is in agreement with uncertainty processing theory (UPT), which suggests that attention may decrease the uncertainty of goal achievement through seeking behavior (Anselme, 2010). It has previously been shown that attention enhances the neural response amplitudes in the brain regions (Lebedev et al., 2004; Baluch and Itti, 2011). Recently, functional brain imaging and neurophysiological studies have reported that the dlPFC is a brain region activated during the processing of visuospatial information and orienting of attention (Bisley and Goldberg, 2010; Balconi and Ferrari, 2012; Katsuki and Constantinidis, 2012; Nazimek et al., 2013), implying an involvement of this region in visual attention.

The present study tested, for the first time to our knowledge, how attention modulates neural responses to unpredictable emotional faces in the dlPFC. The valence asymmetry hypothesis poses that positive emotions are lateralized toward the left and negative emotions toward the right hemisphere (Davidson, 1984; Canli et al., 1998; Beraha et al., 2012; Balconi et al., 2015), especially in prefrontal brain regions (Davidson, 1992; Gur et al., 1994). Building on such valence asymmetry hypothesis, we proposed that attention would modulate neural responses to unpredictable fear faces in right dlPFC (Experiment 1), while it might influence neural responses to unpredictable happy faces in left dlPFC (Experiment 2). A number of recent studies have indicated that unpredictability about future negative emotional events disrupts our ability to avoid them, resulting in amplifying their negative impact (Grupe and Nitschke, 2011, 2013). As the right dlPFC is a brain region linked to negative emotional processing (Davidson, 2002; Nitschke and Heller, 2002; Nitschke et al., 2006), we expected increased activity within the right dlPFC for unpredictable vs. predictable fear faces. It is important to note that a recent study has demonstrated that positive pictures were rated less pleasantly in the unpredictable trials than in the predictable trials (Lin et al., 2012), implying that unpredictability may reduce the positive impact of positive emotional events. It has previously been argued that the left dlPFC serves a more general role in the memory retrieval of positive emotional stimuli (e.g., Balconi and Ferrari, 2012). This seems to suggest that there may be a significantly decreased activation in the left dlPFC for unpredictable compared with predictable happy faces. Interestingly, attention is suggested to contribute to reducing the unpredictability of upcoming emotional events, which decreases individuals' anxiety and fear. Thus, we expected a reversed pattern of results during the attended condition. More specifically, there was diminished right dlPFC activity to unpredictable fear faces and enhanced left dlPFC activity to unpredictable happy faces when these faces were attended.

To test these hypotheses, we adopted a variant of the double-cue paradigm that we employed previously (Chen et al., 2015). In Experiment 1, participants were instructed to perform an emotional task in which fear and neutral target faces were presented randomly, and their blood oxygenation-level dependent (BOLD) brain responses were monitored using 3T functional magnetic resonance imaging (fMRI). In Experiment 2, instead of presenting fear or neutral target faces in Experiment 1, happy or neutral target faces were shown to the participants. A whole brain analysis was performed to identify brain regions showing the impact of attention on unpredictability about upcoming emotional events. In addition, a region of interest (ROI) analysis was conducted to investigate the activation pattern in amygdala, as this region plays a critical role in negative emotion processing (Hamann et al., 2002; Lanteaume et al., 2007).

## Methods

### Participants

Twenty-five healthy volunteers participated in Experiment 1 (14 female, 11 male; mean age = 21.92 years, range = 19–25 years), and 24 healthy volunteers participated in Experiment 2 (12 female, 12 male; mean age = 22.09 years, range = 18–24 years). All participants were right-handed, reported normal or corrected-to-normal vision and had no history of neurological disorder. They gave written informed consent and were financially compensated for their participation. The data were analyzed anonymously, and personally-identifying information was handled confidentially. The study was approved by the local ethics committee and the methods were carried out in accordance with the Helsinki guidelines as per the WHO (Gilder, 1964).

### Stimuli and task

**Figure 1:**
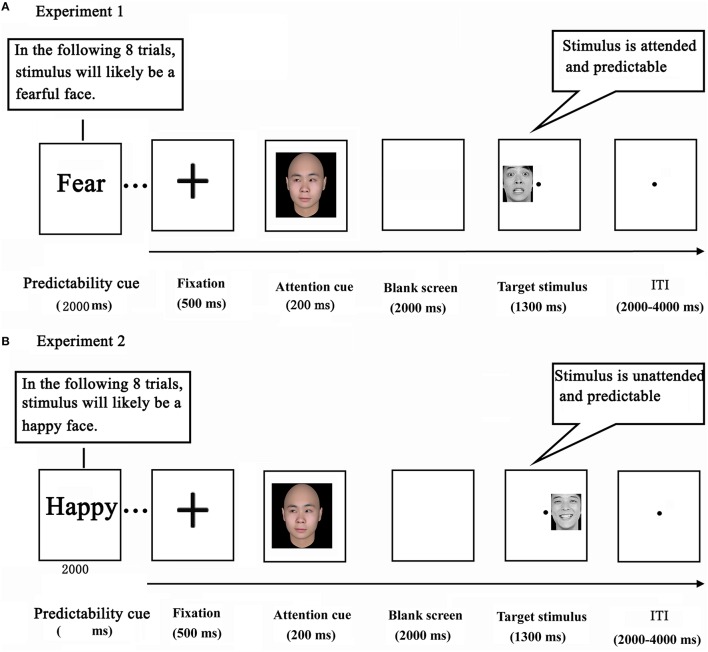
**Experimental design in Experiments 1 (A) and 2 (B)**.

The current study adopted a variant of the double-cue paradigm that we employed previously (Chen et al., 2015). While being scanned with fMRI, the participants performed an emotional task. The task consisted of 21 blocks of 8 trials, yielding a total of 168 trials per participant. Each block of the task was preceded by a predictability cue, which was shown for 2000 ms. In Experiment 1, the predictability cue consisted of either the word “unknown” (an unpredictable condition, containing no information about the emotional expression of target faces) or “fear” (a predictable condition, indicating a 75% likelihood of target faces depicting fear). The predictability cue, in Experiment 2, consisted of either the word “unknown” (a word was identical to the one in Experiment 1), or “happy” (indicating a 75% likelihood of target faces depicting happiness) (Figure 1).

Each trial started with a black cross presented for 500 ms at the center of the screen. Next, a social attention cue was depicted for 200 ms which shifted participants' attention by the direction of gaze. After a delay of 2000 ms, a target stimulus (a face portraying a neutral or fearful expression in Experiment 1, and portraying a neutral or happy expression in Experiment 2) appeared in either the left or the right visual field for 1300 ms (Wang and Luo, 2005). Finally, a blank screen terminated the trial, which lasted for 2000–4000 ms (intertrial interval). Participants were instructed to press the corresponding button as soon as the expression of the target face was detected (response mappings were counterbalanced across participants). Before the actual scan session, participants underwent a practice session in which they performed two to three blocks in order to become familiar with the task. Importantly, the attention cue contained no information about the likely location and expression of a subsequent target face.

### Image acquisition

Magnetic resonance imaging (MRI) data for Experiments 1 and 2 were collected on a 3T Siemens scanner (Siemens Magnetom Trio TIM, Erlangen, Germany). Functional images were acquired by using a gradient echo echo-planar imaging (EPI) sequence (repetition time (TR) = 2000 ms, echo time (TE) = 30 ms, flip angle (FA) = 90°, field of view (FoV) = 192 × 192 mm^2^, matrix size = 64 × 64 pixels, voxel size = 3 × 3 × 3 mm^3^, interslice skip = 0.99 mm, and number of slices = 32). In addition, whole-brain T1-weighted anatomical images (TR = 1900 ms, TE = 2.52 ms, FA = 9°, FoV = 256 × 256 mm^2^) were collected for co-registration prior to three separate functional imaging runs, each lasting for 6 min and 38 s.

### Image analysis

The MRI data in both experiments were preprocessed using SPM8 software package (Friston et al., 1994). The first two volumes of each fMRI scan were discarded to allow for T1 equilibration (Cross et al., 2013; Hortensius and de Gelder, 2014). The remaining 197 functional images were slice-time corrected, and were realigned to the first image to correct for head movements. Next, the anatomical images were co-registered to mean EPI images and segmented into white matter, gray matter and cerebrospinal fluid (CSF). The EPI images were then spatially normalized to the MNI space with the structure information from co-registration and segmentation. Subsequently, the acquired images were spatially smoothed using one 8-mm full-width-at-half-maximum Gaussian kernel.

For Experiment 1, statistical analysis was performed using the general linear model (GLM) implemented in SPM8. On the first level, an event-related design was used, with four types of events: unpredictable fear faces in attended condition (UPAF), unpredictable fear faces in unattended condition (UPUAF), predictable fear faces in attended condition (PAF), predictable fear faces in unattended condition (PUAF). Each event was convolved (time locked to the onset of each target stimulus) with a canonical hemodynamic response function (HRF), and a high-pass temporal filter (cutoff at 128 s) was applied. Each trial was modeled as a separate event (duration = 0). Six regressors representing movement-related variance and one modeling the overall mean were also employed in the design matrix. The first level analysis of each participant yielded four individual contrast images that described the parameter estimates associated with each event modeled. These images were then taken into the second level analysis to construct statistical parametric maps at the group level. F-maps were generated to test for main effects and interactions. According to previous studies (Bell-McGinty et al., 2002; Lee et al., 2010), thresholds of *p* < 0.001 (height) and minimum cluster size *k* > 30 were implemented. The cluster size criterion was used as a conservative measure to minimize false positive activations due to type I errors (Lee et al., 2010). Based on prior hypotheses, small volume corrections were also applied for the dlPFC (Whalley et al., 2012). For the interaction analysis, the average percent signal change was extracted from the significant cluster for each condition using MarsBar (Brett et al., 2002) to examine the direction of the response. All coordinates were reported using MNI convention. For Experiment 2, the statistical analysis was identical to that of Experiment 1, with the exception that an event-related design was adopted at the first level analysis with four types of events (UPAH and UPUAH: unpredictable happy faces in attended and unattended condition, PAH and PUAH: predictable happy faces in attended and unattended condition).

Given that amygdala plays a critical role in negative emotion processing (Hamann et al., 2002; Lanteaume et al., 2007), a region of interest (ROI) analysis was conducted to investigate the activation pattern in the amygdala. It is proposed that memory is associated with top-down predictions (Bar, 2009). We thus used 4 ROIs based on coordinates from a prior fMRI study examining the amygdala function in emotionally influenced memory (Cahill et al., 2004; Table 1). Normalized ROIs with a 5 mm radius sphere centered around these 4 amygdala ROIs (amygdala1–4). Parameter estimates across these ROIs for each condition were extracted. The obtained parameter estimates were then subjected to a 2 × 2 repeated measures ANOVA in SPSS.

**Table 1:** **The coordinates of ROIs**.

**ROIs**	***X***	***Y***	***Z***
amygdala1	22	−12	−15
amygdala2	−18	−12	−15
amygdala3	20	−10	−11
amygdala4	−16	−14	−16

## Results

### Experiment 1: behavioral data

Accuracy and reaction time (RT) measures for each condition were displayed in Table 2. A repeated measures ANOVA with predictability (unpredictable vs. predictable fear faces) and attention (unattended vs. attended) as within-participant factors was calculated on participants' accuracy and RT data.

**Table 2:** **Means and standard deviations of accuracy and reaction time (RT) data for each condition in Experiments 1 and 2**.

**Attention**	**Predictability**	**Accuracy (%)**				**Response time (ms)**			
		**Experiments 1**		**Experiments 2**		**Experiments 1**		**Experiments 2**	
		***M***	***SD***	***M***	***SD***	***M***	***SD***	***M***	***SD***
Attended	Predictable	75.78	15.68	87.58	14.62	727.00	64.20	667.54	63.69
	Unpredictable	78.13	14.21	84.64	17.42	757.03	58.88	693.15	68.21
Unattended	Predictable	78.52	19.63	79.07	18.28	733.74	60.67	694.63	81.16
	Unpredictable	79.33	15.57	84.46	13.19	772.70	59.09	699.44	68.79

Analysis of the accuracy data indicated no significant effects (all *F* < 1.37, NS). With regard to RT data, there was a main effect of predictability [*F*_(1, 24)_ = 32.21, *p* < 0.001, η^2^_*p*_ = 0.573], showing that unpredictable fear faces were processed slower than predictable fear faces. However, there was no significant main effect of attention [*F*_(1, 24)_ = 1.60, *p* =. 218, η^2^_*p*_ = 0.063] or predictability × attention interaction [*F*_(1, 24)_ = 0.85, *p* =. 367, η^2^_*p*_ = 0.034].

### Experiment 1: imaging data

#### Main effect of predictability

The analysis revealed that right middle occipital gyrus (MNI 30 −93 21) and parahippocampal gyrus (MNI 30 0 −18) survived by contrasting predictable fear faces with unpredictable fear faces (see Table 3 for details). No suprathreshold activation was associated with the opposite contrast.

**Table 3:** **Main effect of predictability in Experiments 1 and 2**.

**Region**	**BA**	**Side**	**Voxels**	***Z*-Value**	***P*-Value**	**MNI coordinates**		
						***X***	***Y***	***Z***
**EXPERIMENT 1**								
**Predictable > Unpredictable**								
Middle occipital gyrus	19	R	61	5.04	0.003	30	−93	21
Parahippocampal gyrus	34	R	38	4.48	0.031	30	0	−18
**Unpredictable > Predictable**								
No activated clusters								
**EXPERIMENT 2**								
**Predictable > Unpredictable**								
Postcentral gyrus	3	L	34	4.28	0.035	−66	−12	24
**Unpredictable > Predictable**								
No activated clusters								

*Coordinates (mm) are in MNI space. L, left hemisphere; R, right hemisphere*.

#### Main effect of attention

The contrast testing increased neural activity associated with the attended, relative to the unattended stimuli showed an extensive regions including: left inferior occipital gyrus (MNI −33 −87 −15), right middle occipital gyrus (MNI 27 −99 3), right inferior frontal gyrus (MNI 48 27 6), left insula (MNI −27 24 3) and right posterior cingulate (MNI 3 −27 27) (see Table 4). The reversed contrast yielded significantly more activity in right fusiform gyrus (MNI 21 −60 −6).

**Table 4:** **Main effect of attention in Experiments 1 and 2**.

**Region**	**BA**	**Side**	**Voxels**	***Z*-Value**	***P*-Value**	**MNI coordinates**		
						***X***	***Y***	***Z***
**EXPERIMENT 1**								
**Attended > Unattended**								
Inferior occipital gyrus	18	L	377	5.24	< 0.0001	−33	−87	−15
Middle occipital gyrus	18	R	304	4.65	< 0.0001	27	−99	3
Inferior frontal gyrus	45	R	73	4.39	0.001	48	27	6
Insula	−	L	80	4.26	0.001	−27	24	3
Posterior cingulate	23	R	104	4.24	< 0.0001	3	−27	27
**Unattended > Attended**								
Fusiform gyrus	19	R	133	4.68	< 0.0001	21	−60	−6
**EXPERIMENT 2**								
**Attended > Unattended**								
Middle occipital gyrus	18	R	56	4.29	0.003	24	−96	6
Middle occipital gyrus	18	L	72	4.25	0.001	−45	−81	−3
**Unattended > Attended**								
Lingual gyrus	19	R	52	3.91	0.004	12	−57	−3

Coordinates (mm) are in MNI space. L, left hemisphere; R, right hemisphere

#### Predictability × attention interaction

**Figure 2:**
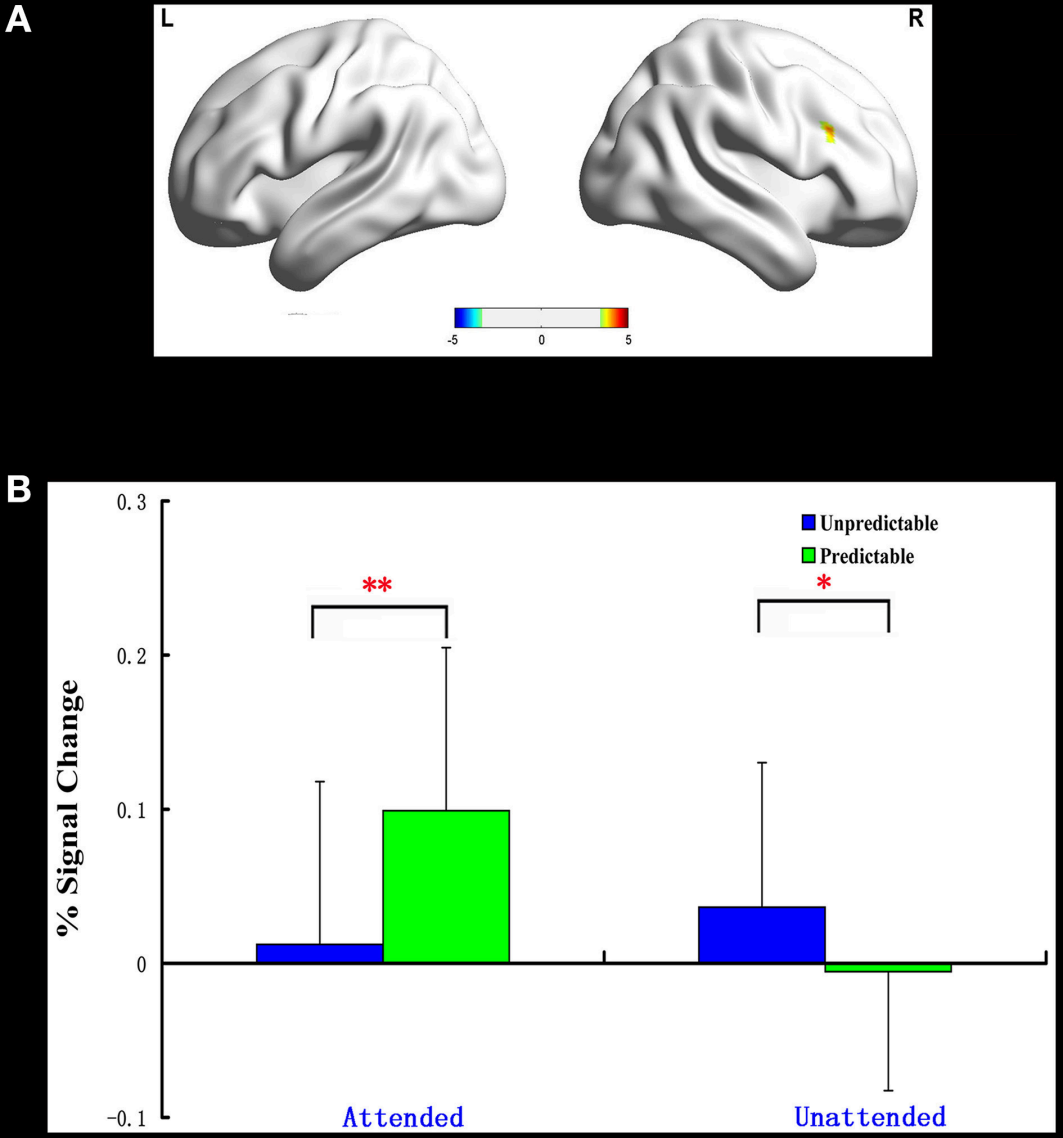
**Interaction of predictability × attention in Experiment 1**. BOLD fMRI activation in the right dlPFC for the two-way interaction **(A)**. The average percent signal change for right dlPFC in each condition **(B)**. The asterisks (^*^) indicate significant differences (^*^p ≤ 0.05, ^**^p ≤ 0.01).

One significant cluster emerged for the interaction of predictability by attention, and it was located in the right dorsolateral prefrontal cortex (MNI 48 21 30, Table 5). The average percent signal change was extracted from this cluster to determine the nature of this interaction. As can be seen in Figure 2, in this region, unpredictable fear faces evoked a larger response than predictable fear faces during the unattended condition (*p* = 0.048). A reversal of pattern, however, was observed during the attended condition (*p* = 0.002).

**Table 5:** **Interaction of predictability × attention in Experiments 1 and 2**.

**Region**	**BA**	**Side**	**Voxels**	***Z*-Value**	***P*-Value**	**MNI coordinates**		
						***X***	***Y***	***Z***
**EXPERIMENT 1**								
**Predictability × Attention**								
Dorsolateral prefrontal cortex	46	R	89	4.15	< 0.0001	48	21	30
**EXPERIMENT 2**								
**Predictability × Attention**								
Dorsolateral prefrontal cortex	46	L	44	4.14	0.011	−51	27	30

*Coordinates (mm) are in MNI space. L, left hemisphere; R, right hemisphere*.

### Experiment 2: behavioral data

Measures of accuracy and RT for each condition can be found in Table 2. There was a significant predictability × attention interaction [*F*_(1, 23)_ = 7.47, *p* < 0.001, η^2^_*p*_ = 0.579]. *Post hoc* tests revealed that unpredictable happy faces were recognized more accurately than predictable happy faces in the unattended condition (*p* = 0.046), but not in the attended condition (*p* = 0.180). For RT data, no main effects and no interaction effects were significant (all *F* < 3.68, NS).

### Experiment 2: imaging data

#### Main effect of predictability

The main effect of predictability on brain activation was displayed in Table 3. The contrast predictable > unpredictable happy faces showed activity of left postcentral gyrus (MNI −66 −12 24). The reverse contrast (unpredictable > predictable happy faces) revealed no statistically significant activity.

#### Main effect of attention

A greater activation of bilateral middle occipital gyrus was observed during attended as compared to unattended condition (MNI 24 −96 6, −45 −81 −3, Table 4). In addition, only the right lingual gyrus showed a higher activation during unattended than attended condition (MNI 12 −57 −3).

#### Predictability × attention interaction

**Figure 3:**
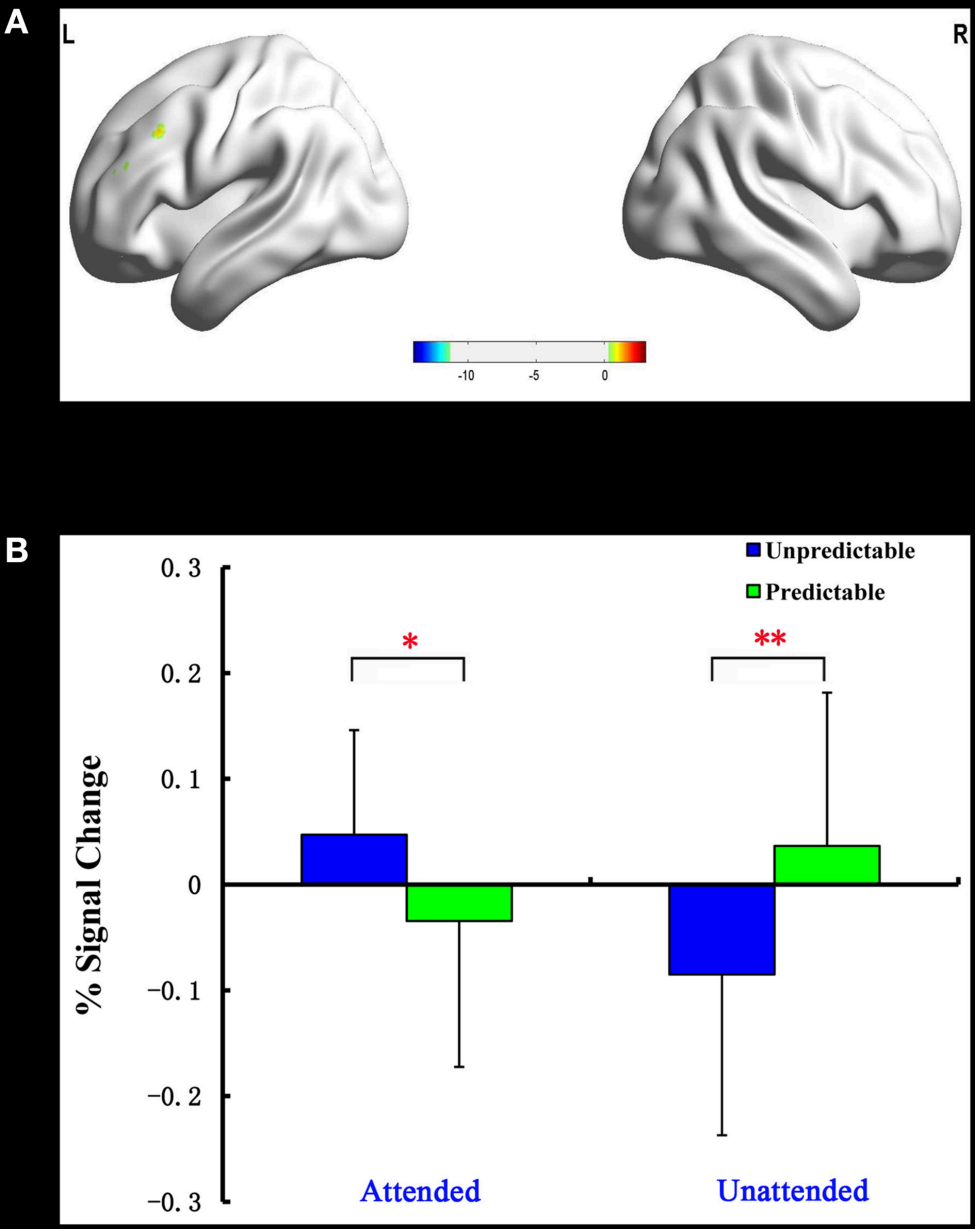
**Interaction of predictability × attention in Experiment 2**. BOLD fMRI activation in the left dlPFC for the two-way interaction **(A)**. The average percent signal change for left dlPFC in each condition **(B)**. The asterisks (^*^) indicate significant differences (^*^p ≤ 0.05, ^**^p ≤ 0.01).

The predictability × attention interaction revealed a significant activation cluster in the left dorsolateral prefrontal cortex (MNI –51 27 30), as illustrated in Table 5. The average percent signal change in this cluster for each condition was shown in Figure 3. There was a significantly reduced activation for unpredictable compared with predictable happy faces during the unattended condition (*p* = 0.010), while there was a significantly enhanced activation for unpredictable relative to predictable happy faces during the attended condition (*p* = 0.039).

## ROI analysis results

ROI analysis showed that there was a significantly increased activation in the amygdala2 for unpredictable relative to predictable fear faces during the unattended condition (*p* = 0.038). While there was a trend of decreased activation in this region for unpredictable vs. predictable fear faces during the attended condition, the result was Non-significant (*p* = 0.108). Unlike the amygdala2, no other amygdala ROIs exhibited significant activity.

## Discussion

The present work employed a variant of the double-cue paradigm to investigate how attention modulated neural responses to unpredictable emotional faces. Behaviorally, we reported a fear-unpredictable effect whereby participants responded slower to unpredictable faces than predictable faces in Experiment 1. In Experiment 2, a happy-unpredictable effect with higher accuracy to unpredictable happy faces than predictable happy faces was observed when these faces were unattended. The fMRI results of Experiment 1 showed that the right dlPFC expressed increased activation for unpredictable relative to predictable fear faces during the unattended condition. A reversal of pattern, however, was observed during the attended condition. Unlike the fMRI results for Experiment 1, those of Experiment 2 disclosed a significantly reduced activation in the left dlPFC for unpredictable compared with predictable happy faces during the unattended condition and a significantly enhanced activation in this region for unpredictable relative to predictable happy faces during the attended condition. Finally, the ROI analysis showed a significantly increased activation in the amygdala2 for unpredictable vs. predictable fear faces during the unattended condition.

The current study revealed a fear-unpredictable effect at the behavioral level, which suggests that participants were more cautious for unpredictable fear faces. It could be the case that unpredictability about future fear events increased individuals' anxiety and fear (e.g., Sarinopoulos et al., 2009; Grupe and Nitschke, 2011, 2013; Jin et al., 2013; Lin et al., 2014). Moreover, a happy-unpredictable effect with higher accuracy to unpredictable faces than predictable faces was found when these faces were unattended. However, such happy-unpredictable effect vanished when the faces were attended, probably reflecting that attention may reduce the unpredictability of upcoming happy faces (Anselme, 2010).

### Attention modulates neural responses to unpredictable fear faces in right dlPFC

It has previously been argued that the right dlPFC is thought to be associated with negative emotion processing (Davidson, 2002; Nitschke and Heller, 2002; Nitschke et al., 2006). The present fMRI data showed that the neural responses to unpredictable fear faces were enhanced in right dlPFC compared to predictable fear faces when these faces were unattended, implying that unpredictability about potential negative events may amplify the negative impact of these events. This viewpoint is supported and documented by a number of previous studies examining emotional unpredictability (e.g., Sarinopoulos et al., 2009; Grupe and Nitschke, 2011, 2013; Lin et al., 2014). A similar pattern of activation has been found in previous fMRI studies which addressed ambiguity within different paradigms, such as decision-making (Huettel et al., 2006; Bach et al., 2009; Shackman et al., 2009). Indeed, those fMRI studies have revealed that the right dlPFC is involved in encoding decision making about ambiguous outcomes (Huettel et al., 2006; Bach et al., 2009).

A different pattern of results was observed for attended stimuli. When stimuli were attended, the neural responses in right dlPFC were reduced for unpredictable compared with predictable fear faces. Using functional imaging, it has been shown that there is a positive correlation between right dlPFC neural activity and self-reports of negative affect (Nitschke et al., 2006; Ochsner and Gross, 2008). The right dlPFC hypoactivity observed here thus indicates that attention might reduce the amplifying negative impact caused by unpredictability about future fear faces. One potential explanation is that attention may help to detect relationships between specific cues and subsequent events since it actively in prove the precision of inference (Rao, 2005; Friston, 2009). Such contingency detection allows individuals to explain past events and more appropriately prepare for the future (Sarinopoulos et al., 2009; Grupe and Nitschke, 2013), which is suggested to result in the reduction of unpredictability about incoming fear faces, and ultimately decreases individuals' anxiety and fear.

### Attention influences neural responses to unpredictable happy faces in left dlPFC

Here, it is interesting that we observed a lower left dlPFC activation for unpredictable relative to predictable happy faces during the unattended condition. There has been a growing recognition that the left dlPFC serves a more general role in the memory retrieval of positive emotional stimuli (e.g., Balconi and Ferrari, 2012). This seems to suggest that the reduction in activity may reflect that unpredictability about upcoming happy faces reduces the positive impact of these faces, which fits well with the findings in clinical depression. Previous studies found that patients with depression, a condition that affects the ability to detect and take pleasure in future events, showed specific decreases in glucose metabolic activity in their left dlPFC (Baxter et al., 1989; Rajkowska et al., 2001).

However, more left dlPFC activity for unpredictable compared with predictable happy faces was found during the attended condition. According to the role of the left dlPFC described above (Balconi and Ferrari, 2012), our finding concerning enhanced activity in the left dlPFC suggests that attention might improve the decreased positive impact of unpredictable happy faces. This provides further evidence for a specific role of attention in the reduction of unpredictability about future emotional events. More specifically, attention may contribute to reducing the unpredictability about incoming happy faces, increasing the pleasure that people derive from these unpredictable happy faces. A similar finding was reported recently by Kay et al. (2015), who suggested that attention reduced spatial uncertainty in human ventral temporal cortex.

As unpredictability may increase individuals' anxiety and fear (Grupe and Nitschke, 2013; Lin et al., 2014), one would predict increased activity in the amygdala for unpredictable vs. predictable faces. This is corroborated by our subsequent ROI analysis, which revealed significantly enhanced activity in the amygdala2 for unpredictable relative to predictable fear faces during the unattended condition. Previous studies examining social anxiety have also demonstrated that the amygdala is recruited under conditions of unpredictability (Lorberbaum et al., 2004; Guyer et al., 2008). For example, Lorberbaum et al. (2004) reported heightened amygdala activity when clinically anxious children anticipated unknown peer feedback.

Like other studies, the present study is not without limitations. Given that neutral faces are not always perceived as neutral, one limitation is that this study separated the facial expressions into two different paradigms, since. It should present all of the facial expressions within the same paradigm.

## Conclusion

While a wealth of research has documented the neural correlates of unpredictability or uncertainty (Brown et al., 2008; Bach et al., 2009; Lin et al., 2014), there has been no study to date that directly investigates how attention affects the neural mechanisms for unpredictable emotional faces. At the behavioral level, the present study reported a fear-unpredictable effect. Additionally, a happy-unpredictable effect, which was different from the fear-unpredictable effect, was observed only when these faces were unattended. On the neural level, during the unattended condition, we observed increasing right dlPFC activity for unpredictable fear faces and decreasing left dlPFC activity for unpredictable happy faces. This indicated that unpredictability may amplify the negative impact of fear faces and reduce the positive impact of happy faces (Rajkowska et al., 2001; Grupe and Nitschke, 2013). A reversed pattern of results, however, was found during the attended condition. More specifically, there was diminished right dlPFC activity to unpredictable fear faces and enhanced left dlPFC activity to unpredictable happy faces when these faces were attended. We suggest that attention may be critical to reducing the unpredictability about future emotional events (Friston, 2005, 2009; Grupe and Nitschke, 2013). Our findings contribute to a better understanding of the neural mechanisms of unpredictability about future emotional events and the growing body of literature exploring the resolution of unpredictability.

## Author contributions

GR, XC, and QZ designed the experiments. GR collected and analyzed the data. QZ and YM assisted with the experimental setup. GR and XC primarily wrote the manuscript. All authors discussed the results and commented on the manuscript.

### Conflict of interest statement

The authors declare that the research was conducted in the absence of any commercial or financial relationships that could be construed as a potential conflict of interest.
